# Lewis and Clark Dental Congress, Portland, Oregon

**Published:** 1905-03

**Authors:** 


### Editorial.

### LEWIS AND CLARK DENTAL CONGRESS, PORTLAND, OREGON.

It is difficult to realize the rapidity with which countries, cities, and peoples develop from primeval conditions to the civilization that marks the progress of the twentieth century. Persons are now living who can remember before Chicago was incorporated into a city, 1837, and when St. Louis was the last fitting-out city for expeditions over the plains, and Oregon the last place on the map where a civilized man would desire to locate. The imagination of the writer was vividly impressed in his youthful years with the published reports of the Lewis and Clark expedition and the subsequent efforts of Frémont and others to solve the problems connected with the great divide which the Rocky Mountains made between the East and the West. The last remnants of the aborigines were a constant source of interest, and the trapper and his perils in his lonely quest for fur were more vividly impressed on the boyish imagination than the dry details of ancient and modern history.

It is, therefore, difficult to realize that the civilization of the past has been transplanted to the Pacific coast, fully armed with all the essentials to meet the demands of an exacting age, and with more of the vigor than is manifested in the older sections.

We have hardly begun to experience a recovery from the strain of the International Dental Congress, held at St. Louis, when word is received that at Portland, Ore., will be assembled, July 17, 18, 19, and 20, 1905, the Lewis and Clark Dental Congress, and if it be possible to judge from the advanced sheets detailing plans, this will be one of the most interesting of the many congresses held, not excepting those of an international character. The preliminary work thus far accomplished indicates this, and promises to those prepared to attend a treat not often vouchsafed to the weary workers in dental conventions of the cut-and-dried character.

Upon another page is presented the names of the officers and general committees, a sufficient guarantee for the success of the Congress.

While membership in this congress requires a recommendation from a member of the General Committee for those living west of the Rocky Mountains, this is not required of those applying for membership from the East. These applications are to be sent direct to the Secretary, to be acted upon by the Executive Committee. The membership fee for the Congress is fixed at five dollars. The officers are very anxious that the representation should be large from the East, and this desire will, without doubt, be seconded by many who will arrange to be present, and the moderate rates from the Middle West should certainly assure an equal reduction from other points, and a large delegation from the eastern sections of the country.

A letter in relation thereto from the Secretary states that "Railroad rates to Portland, Ore., during the Lewis and Clark Fair will be fifty-six dollars for the *round trip from Chicago*, and if dentists residing east of the Missouri River will, instead of taking their vacation in the mountains or in Europe, embrace this opportunity to join with us in the Lewis and Clark Dental Congress, we can show them, besides an interesting professional and scientific programme, a country of exceeding beauty. The tickets will be on sale at all the railroad ticket-offices. The routes may be selected, both coming and going, by the purchaser, and the tickets will be good for ninety days for the round trip. . . . Returning east they can visit Yellowstone Park, Alaska, Puget Sound, see the Columbia River from Portland, and can visit the Yosemite Valley, San Francisco, and Southern California.

"We expect an attendance in the territory west of the Rocky Mountains of at least one thousand men, and, in order to make the programme interesting, are desirous of securing essayists and clinicians among prominent men of the Atlantic coast."

It will thus be seen that the interest involved, outside of the Congress, should be sufficient to attract the professional man confined day by day, and month by month, in one apartment. Those who can take this trip are to be envied by their less fortunate brothers, for it is one that comprises more of real interest to the traveller, in its topographical features, than probably any other that could be taken in the broad domain of the United States.

It seems, aside from this, that something is due the dentists of the Western coast. They have contributed more than their share in making our Eastern meetings interesting, and now that they have a congress worthy of a great effort, let all who can join hands with them and demonstrate that, though leagues separate, professionally and fraternally we are one in spirit and practice.

